# Development of a chiral HPLC method for the separation and quantification of hydroxychloroquine enantiomers

**DOI:** 10.1038/s41598-021-87511-5

**Published:** 2021-04-13

**Authors:** Xisheng Xiong, Kun Wang, Tao Tang, Jinzhi Fang, Yijun Chen

**Affiliations:** State Key Laboratory of Natural Medicines and Laboratory of Chemical Biology, China Pharmaceutical University, 639 Longmian Avenue, Jiangning District, Nanjing, 211198 China

**Keywords:** Drug discovery, Diseases

## Abstract

Hydroxychloroquine (2-[[4-[(7-Chloroquinolin-4-yl) amino]pentyl](ethyl) amino]-ethanol, HCQ), an effective anti-malarial drug, has been tested in the clinics for potential treatment of severe coronavirus disease 2019 (COVID-19). Despite the controversy around the clinical benefits of HCQ, the existence of a chiral center in the molecule to possess two optical isomers suggests that there might be an enantiomeric difference on the treatment of COVID-19. Due to their poor resolution and the inability of quantification by previously reported methods for the analysis of HCQ enantiomers, it is necessary to develop an analytical method to achieve baseline separation for quantitative and accurate determination of the enantiomeric purity in order to compare the efficacy and toxicity profiles of different enantiomers. In this study, we developed and validated an accurate and reproducible normal phase chiral high-performance liquid chromatography (HPLC) method for the analysis of two enantiomers of HCQ, and the method was further evaluated with biological samples. With this newly developed method, the relative standard deviations of all analytes were lower than 5%, and the limits of quantification were 0.27 μg/ml, 0.34 μg/ml and 0.20 μg/ml for racemate, R- and S-enantiomer, respectively. The present method provides an essential analytical tool for preclinical and clinical evaluation of HCQ enantiomers for potential treatment of COVID-19.

## Introduction

As severe coronavirus disease 2019 (COVID-19) pandemic progresses, effective therapeutics are urgently needed to ease the outbreak and save more lives^1–5^. In addition to the anti-malarial use, hydroxychloroquine (HCQ) has been utilized for the treatment of systemic lupus erythematosus^6, 7^, rheumatoid arthritis^8^, primary Sjogren's syndrome and other diseases. Based on in vitro evidence of inhibiting the replication of HIV-1^9, 10^ and COVID-19 virus^11–14^, HCQ has been thought to be effective in clinical settings to combat COVID-19 pandemic. However, the results of various clinical trials of using HCQ for COVID-19 have been contradictory^15–19^, due mainly to the differences on infection stage and dosing. Meanwhile, HCQ exhibits serious side effects, especially cardiac and retinal toxicities^20–25^. On the other hand, chloroquine, a structural analogue of HCQ, has recently been reported to be beneficial for reducing the length of hospital stay and postponing the deterioration of COVID-19 infected patients^26^. Given the structural similarity, same mode of action and less toxic, HCQ has drawn significant attention for its clinical usefulness for the treatment of COVID-19, and several large-scale multicenter clinical trials have been scheduled^27^.

**Figure 1:**
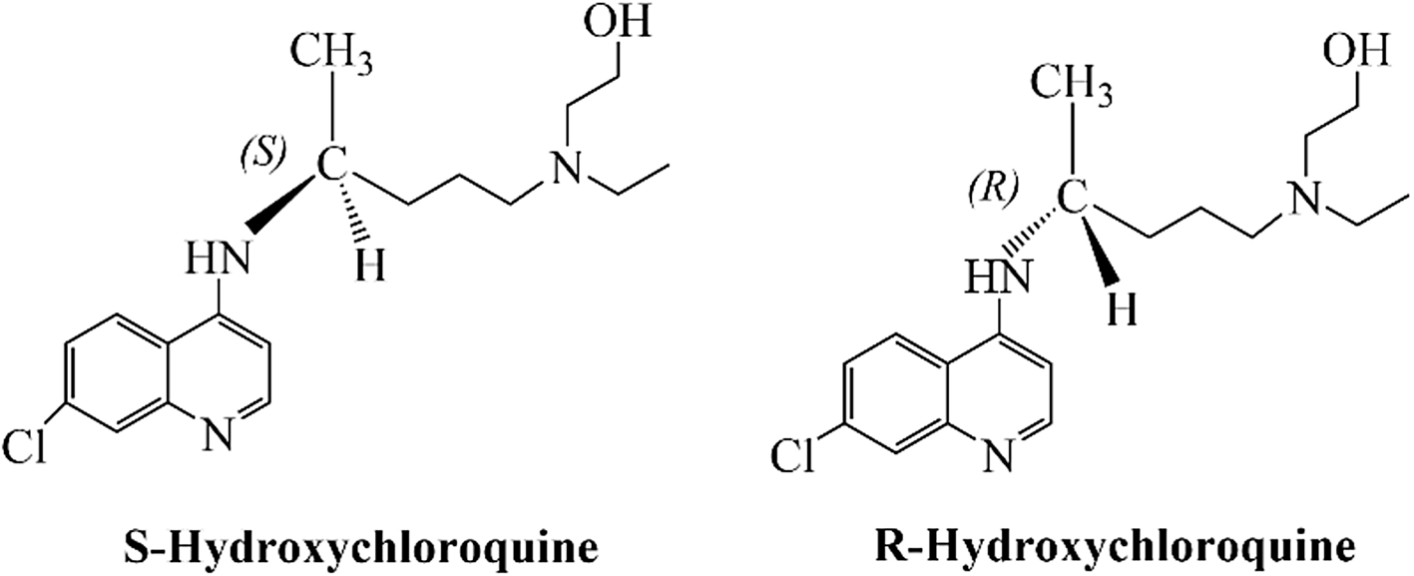
Chemical structures of hydroxychloroquine enantiomers.

Although HCQ was approved for clinical use as a racemate, the existence of a chiral center in HCQ may result in enantiomeric difference on the efficacy and toxicity. Indeed, previous metabolic studies have shown that the concentration of (R)-(−)-HCQ in blood was 1.6–2.9 times that of (S)-(+)-HCQ (Fig. 1), and renal clearance rate of (S)-HCQ was approximately twice that of (R)-enantiomer^20^, strongly indicating that two enantiomers may exhibit different profiles of efficacy, toxicity and metabolic preference. Recently, the chiral switches of HCQ and/or CQ for the treatment of COVID-19 have been proposed, suggesting that S-HCQ would be more active and safer than R-enantiomer for the possibility of higher dosing and/or longer period of administration^28^. Therefore, the comparison of HCQ enantiomers on the efficacy and toxicity for treating COVID-19 would clarify the differences and provide more effective and safer therapeutic option for this global health crisis.

To compare biological activities and therapeutic values of HCQ enantiomers, the separation of the enantiomers and the analysis of the enantiomeric purity are a prerequisite. In 1991, α_1_-acid glycoprotein (AGP) stationary phase was used for the first time to separate the enantiomers of HCQ and their major metabolites. Although partial separation was achieved, it was far from baseline resolution, not useful for the quantification of enantiomeric purity^29^. Later, chiral-AGP column was used for the separation of HCQ enantiomers with a mobile phase consisted of sodium phosphate buffer-ethanol-acetonitrile and *N*,*N*-DMOA as an additive. With this system, the resolution between HCQ enantiomers was claimed to be 2.05, but two enantiomers were actually overlapped above the baseline in the chromatograms^30^. Subsequently, chiral-AGP column was used to couple a modified buffered mobile phase for the separation of HCQ enantiomers and their metabolites. Although the resolution of HCQ enantiomers was reported to be 3.2 with this method, the actual situation was not baseline separation^31^, still unable to quantify the enantiomeric purity. A decade later, two groups of researchers reported Chiralpak-AD-based methods for the analyses of HCQ enantiomers and their metabolites to investigate the enantiomeric preference on drug metabolism. Unfortunately, in addition to the reproducibility issue, the reported resolution of HCQ enantiomers was very poor, which was unable to accurately and quantitatively analyze the actual values of respective enantiomer of HCQ^32, 33^.

To achieve baseline separation and reproducibly analyze HCQ enantiomers, a reliable and accurate analytical method is obviously required for potential chiral switch. In the present study, we developed a Chiralpak AD-H based normal phase HPLC method after systematic optimization of the chromatographic conditions. After validation of the analytical method, we further applied this method for the determination of plasma concentration of HCQ enantiomers. This study provided a reliable, reproducible and quantitative method for the analysis of individual enantiomers of HCQ, which could be used for preclinical and clinical studies on the enantiomeric differences, developing single enantiomer as a potential new therapy for COVID-19 pandemic.

## Results and discussion

### Optimization of chromatographic conditions with chiralpak AD-H column

Despite the poor resolution of HCQ enantiomers by reported method (Fig. S4), little separation of the enantiomers was observed under normal phase conditions. Combined this observation and the failure of using reversed phase chiral columns, we therefore decided to optimize chromatographic conditions based on Chiralpak AD-H column.

Firstly, the influence of the ratio of n-hexane and isopropanol on the resolution and retention was examined at 90:10, 90.5:9.5, 91:9, 91.5:8.5, 92:8, 92.5:7.5, 93:7, respectively. With the decrease of isopropanol, the retention of the enantiomers on the column was prolonged, and the separation was also better (Fig. S2), which is in agreement with the general phenomena. To balance the resolution and analysis time, we choose the ratio of 93:7 (hexane–isopropanol) as mobile phase for further optimization.

Since HCQ is a basic molecule containing multiple nitrogen atoms for ionization, the addition of a basic additive in the mobile phase could improve the resolution of the enantiomers. Thus, we compared two commonly used basic additives, diethylamine (DEA) and triethylamine (TEA). We found that the effects on the resolution by DEA were more obvious (data not shown), which is the same as previous reports^32, 33^. Consequently, we examined the influence on the resolution by different amounts of DEA. As shown in Figure S3, there was no linear relationship between DEA concentration and resolution. However, a definitive trend was observed, in which the increase of DEA correlated with better resolution and 0.35% DEA in n-hexane showed the best resolution at 25 °C. When column temperature was considered, the resolution of 0.5% DEA in n-hexane (Rs = 2.08) at 20 °C was obviously better than that of 0.35% DEA (Rs = 1.71). As a result, we decided to add 0.5% DEA in n-hexane with a column temperature of 20 °C.

**Figure 2:**
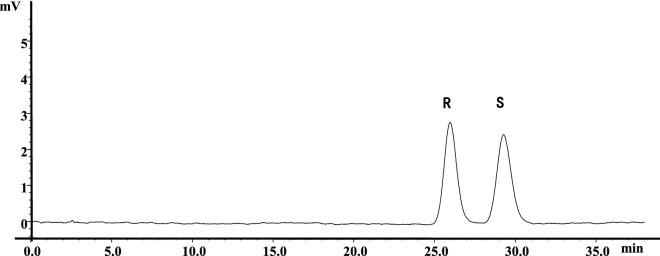
Chromatogram for the resolution of racemic HCQ. Chromatographic conditions: Chiralpak AD-H (4.6 mm × 150 mm, 5 μm particle size); n-hexane-isopropanol (93:7, v/v) plus 0.5% DEA into hexane as mobile phase at a flow rate of 0.8 ml/min with UV detection at 343 nm at 20 °C.

Due to the fact that chiral columns commonly require relatively slower flow rates, we next evaluated the effects of flow rate on the separation of HCQ enantiomers with established analytical method. Although slower flow rates usually generate broad peaks and longer analysis time, the decrease of flow rate from 1.0 to 0.8 ml/min resulted in a satisfactory resolution and reasonable analysis time, in which complete baseline separation of the enantiomers was achieved. Therefore, the optimized chromatographic conditions on Chiralpak AD-H column were the use of mobile phase system consisting n-hexane containing 0.5% DEA and isopropanol at the ratio of 93:7 (v/v) with the flow rate of 0.8 ml/min at 20 °C. Under the optimal conditions, typical retention times of (R)-HCQ and (S)-HCQ were found to be 26 and 29 min with a resolution value of 2.08 (Fig. 2), which could accurately quantify the enantiomeric purity of two enantiomers based on their peak areas.

### Preliminary validation of chiralpak AD-H based method

The linear regression curves between peak area and sample concentration were obtained for Rac-HCQ samples ranging from 1 to 25 μg/ml. Three standard curves of Rac-, S- and R-HCQ were plotted respectively, and the linear equations and the square values of the correlation coefficient (R^2^) were obtained to show excellent correlation (R^2^ > 0.995, Supplementary Equation). Meanwhile, from the comparison of three different batches of Chiralpak AD-H column, no significant variations, in terms of resolution, retention time and peak area, were observed.

**Figure 3:**
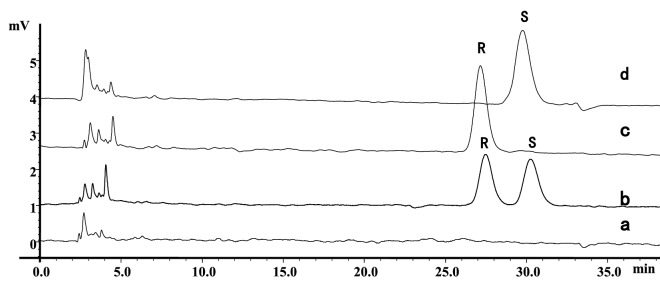
Comparison of chromatograms of blank rat plasma and samples incubated with rat plasma. Same analytical conditions were used as Fig. 1. (**a**) Blank; (**b**) 15 μg/ml of Rac-HCQ-sulfate incubated with rat plasma; (**c**) 10 μg/ml of (R)-HCQ-sulfate incubated with rat plasma; and (**d**) 10 μg/ml of (S)-HCQ-sulfate incubated with rat plasma.

Using formula (1) and (2) in the section of Materials and methods, we calculated the values of LOQ and LOD for racemate, S- and R-HCQ. The LOQ and LOD for racemate were 0.27 μg/ml and 0.09 μg/ml; the LOQ and LOD for S-HCQ were 0.20 μg/ml and 0.07 μg/ml; and the LOQ and LOD for R-HCQ were 0.34 μg/ml and 0.11 μg/ml. In addition, the specificity of this analytical method was verified by comparing plasma samples between blank and different optical isomers of HCQ (Fig. 3).

The accuracy and precision of the method were examined using linear equations. Table 1 summarizes the results of the samples tested on the same day, 1 day apart and 4 days apart. In all cases, intra-day (0 day) and inter-day (1 day) variability were lower than 5%. After 4 days, the variability compared to day 1 was above 5%. These data indicated that the present method is highly reliable and reproducible. On the other hand, the data also suggested that the samples are not suitable to store at room temperature for longer than 24 h at which the concentrations were remarkably higher than the beginning, due mainly to the volatility of the sampling solvent.

**Table 1:** Precision and accuracy of the method for the analysis of HCQ and its enantiomers.

Time/d	Theoretical concentration		Obtained concentration ± SD*	R.S.D^a^
	(μg/ml)		(μg/ml)	%
0	Rac^b^	10	9.67 ± 0.10	1.01
	R^c^		9.58 ± 0.12	1.25
	S^d^		9.76 ± 0.10	1.05
1	Rac	10	10.48 ± 0.41	3.90
	R		10.69 ± 0.40	3.78
	S		10.69 ± 0.19	1.76
	Rac	15	15.98 ± 0.36	2.24
	R		15.70 ± 0.86	2.15
	S		15.93 ± 0.37	2.35
	Rac	20	21.93 ± 0.43	1.98
	R		21.72 ± 0.26	1.18
	S		22.15 ± 0.61	2.77
4	Rac	10	10.77 ± 0.36	3.30
	R		10.88 ± 0.33	3.01
	S		10.93 ± 0.08	0.75
	Rac	15	16.18 ± 0.82	5.07
	R		16.17 ± 0.71	4.39
	S		16.18 ± 0.93	5.74

*n = 3; ^a^relative standard deviation; ^b^racemic HCQ; ^c^R-enantiomer; ^d^S-enantiomer.

### The advantages of the present method

Compared to previously reported methods^29–33^, the present method collectively possesses a number of advantages: (a) complete baseline separation between two enantiomers of HCQ has been achieved; (b) quantitative analysis of respective enantiomer has been enabled; (c) isocratic elution has been conducted without using salts in the mobile phase; and (d) excellent specificity and reproducibility have been demonstrated. Consequently, the present method could not only accurately assess the optical purity of HCQ, but also potentially be useful for quantitative determination of single enantiomer in biological samples.

## Conclusion

In this study, a simple and accurate normal phase chiral HPLC method was developed for the enantiomeric separation and quantification of S- and R-HCQ. The baseline separation of two enantiomers allowed to quantitatively determine the concentrations of HCQ enantiomers, which was preliminarily validated for its accuracy, precision, sensitivity, specificity and reproducibility. The present method can be applied to quantitate HCQ enantiomers for chiral switch as well as pharmacokinetic and toxicokinetic studies, which could be useful to thoroughly assess and compare the efficacy and toxicity profiles of HCQ enantiomers. More importantly, the present method will be a valuable tool to assist the development of potential therapeutics for the treatment of COVID-19.

## Material and methods

### Chemicals

Racemic hydroxychloroquine (Rac-HCQ) sulfate, (R)-(–)-HCQ sulfate and (S)-(+)-HCQ sulfate were from Desite Biopharmaceutical Co., Ltd (Chengdu, China). HPLC grade n-hexane was purchased from Aladdin (Shanghai, China). Isopropanol was from Sinopharm Chemical Reagent Co., Ltd (Shanghai, China). Methanol and acetonitrile were from Tedia (Fairfield, USA). Diethylamine (DEA) was obtained from Ling Feng Chemical Reagent Co., Ltd (Shanghai, China).

### Animal welfare and ethics

All animal experiments were performed in accordance with the relevant laws and regulations on the use and management of experimental animals. The number of animals, the design of tests and the disposal of animals were approved by the Animal Care and Use Committee of Center of New Drug Safety Evaluation and Research at China Pharmaceutical University with the approval number of B20200515-1 and were strictly carried out. The animal studies were conducted in compliance with the ARRIVE guidelines.

### Optimization of chiral HPLC conditions

HPLC analyses were performed on a LC-2010A HT (Shimadzu, Kyoto Japan) consisting of an UV–Vis detector, automatic sampler and thermostatic column oven compartment. The resolution of HCQ enantiomers was achieved on a Chiralpak AD-H column (4.6 mm × 150 mm, particle size 5 μm) with column oven maintained at 20 °C. The mobile phase consisted: (A) n-hexane in the presence of 0.5% DEA, and (B) isopropanol. Two portions of mobile phases were mixed online (93:7, v/v) with an isocratic elution at a flow rate of 0.8 ml/min. The UV wavelength at 343 nm was set for detection with an injection volume of 10 μl.

### The influence of diethylamine (DEA) on the resolution

The influence of DEA added in the mobile phase on the resolution of HCQ enantiomers was investigated under the optimal chromatographic conditions with Chiralpak AD-H column at 25 °C. The content of DEA in n-hexane was 0.0%, 0.10%, 0.15%, 0.20%, 0.25%, 0.30%, 0.35%, 0.40%, 0.45%, 0.50%, respectively.

### Sample preparations

The stock solutions of Rac-HCQ sulfate, (R)-HCQ sulfate and (S)-HCQ sulfate were prepared at 1 mg/ml in water. Working solutions of Rac-HCQ sulfate at concentrations of 1, 5, 10, 15 and 20 μg/ml were prepared by dilution with water. Working solutions of (R)-HCQ sulfate and (S)-HCQ sulfate were prepared at 10 μg/ml by dilution with water respectively. All solutions were temporarily stored at 4 °C before the analysis.

### Preparation of plasma samples

Drug-free blood (~ 12 ml) from the abdominal aorta of healthy SD rats was taken in a vacuum sampling vessel containing EDTA-K_2_, and then centrifuged at 3000 rpm for 10 min at 4 °C to obtain the plasma (~ 5 ml). In 10 ml centrifuge tube, 500 μl plasma was mixed with 500 μl working solution of racemate and enantiomers of HCQ supplemented with 250 μl potassium phosphate (pH 7.4, 50 mM) and 250 μl 2% sodium bicarbonate. The above solution was mixed and incubated at room temperature for 1 h before the addition of 1.5 ml cold acetonitrile and 2 μl of 5 N sodium hydroxide (pH 10.0). After incubation for 1 h, 3 ml chloroform was added for extraction. The extracted sample was centrifuged at 3000 rpm for 5 min and the organic layer separated and evaporated by nitrogen gas. The residue was dissolved in 1 ml of hexane–isopropanol (93:7, v/v, containing 0.5% DEA).

### Method validation

The optimized method for Rac-HCQ and two enantiomers were further validated preliminarily. The factors for the examination included accuracy, precision (repeatability and intermediate precision), sensitivity (limit of quantification and limit of detection) and specificity.

Calibration standards of Rac-HCQ at 1, 5, 10, 15, 20, 25 μg/ml, and the concentrations of each enantiomer at half of these were freshly prepared by dilution of stock solution into the mobile phase, and the mobile phase without HCQ was used as blank. The theoretical concentrations of Rac-HCQ (free base) were 0.77, 3.87, 7.74, 11.61 and 15.48 μg/ml. Plots of the concentrations of Rac-HCQ and two enantiomers versus peak area were generated, and the equations of linear regression were applied to the determination of the concentrations of racemate and enantiomers respectively.

The sensitivity of the method was assessed by identifying the limit of quantification (LOQ). Residual standard deviation (σ) method was implemented to predict the values of LOQ and limit of detection (LOD) by following formula (1) and (2) and the precision was established at these predicted levels. The specificity of the method was verified by comparison of blank and sample solutions.

1 $$LOQ=10\sigma /S$$

2 $$LOD=3.3\sigma /S$$

where σ = residual standard deviation of response; S = slope of the calibration curve.

The accuracy and precision of this method were evaluated by same day (n = 3) and different days (n = 3) using Rac-HCQ (free base) at the concentrations of 10, 15 and 20 μg/ml, and the concentrations corresponding to each enantiomer were half of these concentrations. The results are shown by relative standard deviation (R.S.D).

## Supplementary Information

Supplementary Information 1.
