# Supplementary material for: Development of a chiral HPLC method for the separation and quantification of hydroxychloroquine enantiomers
Source: Sci Rep. 2021 Apr 13;11:8017. doi: 10.1038/s41598-021-87511-5 (PMC8044086; doi:10.1038/s41598-021-87511-5)
Supplement: Supplementary file 1 — Supplementary Information 1. [file 41598_2021_87511_MOESM1_ESM.docx]

Supplementary Information for

Development of a chiral HPLC method for the separation and quantification of hydroxychloroquine enantiomers

Xisheng Xiong, Kun Wang, Tao Tang, Jinzhi Fang, Yijun Chen^1*^

^1^State Key Laboratory of Natural Medicines and Laboratory of Chemical Biology, China Pharmaceutical University, 639 Longmian Avenue, Jiangning District, Nanjing, 211198, China

^*^ Corresponding author. [yjchen@cpu.edu.cn](mailto:yjchen@cpu.edu.cn)

**This file includes:**

I. Supplementary Methods

II. Supplementary Figures

III. Supplementary Equations

IV. Supplementary Results

**I. Supplementary Methods**

To determine chemical purity of Rac-HCQ, YMC-pack ODS-A column (250×4.6 mm I.D.S, 12 nm) was used at flow rate of 1.0 ml/min at 40 ℃ with Agilent 1260 HPLC instrument. The elution procedure were as follow: 0 min, 10% B (acetonitrile containing 0.1% TFA) -90% A (water containing 0.1% TFA); 0-2 min: 35% B-65% A; 2-8 min: 40% B-60% A; 8-10 min: 90% B-10% A; 10-13 min: 10% B-90% A. With this elution procedure, the separation of two enantiomers of Rac-HCQ by RP chiral columns was initially performed with different compositions of mobile phase.

Reversed-phase chiral columns, including Chiralcel OJ-RH, OX-RH, OZ-RH and Chiralpak AD-RH, AS-RH, AY-RH, AZ-RH, were used to examine the separation of HCQ enantiomers with gradient elution at a flow rate of 0.5 ml/min. The mobile phase consisted: (A) water in the presence of 0.1% TFA, and (B) acetonitrile in the presence of 0.1% TFA. Other gradient elution procedures examined by RP chiral columns are listed as follows.

(1)

| Time/min | A% | B% |
| --- | --- | --- |
| 0 | 90 | 10 |
| 30 | 10 | 90 |
| 35 | 90 | 10 |

(2)

| Time/min | A% | B% |
| --- | --- | --- |
| 0 | 90 | 10 |
| 5 | 90 | 10 |
| 20 | 85 | 15 |
| 30 | 75 | 25 |
| 40 | 50 | 50 |
| 45 | 90 | 10 |

(3)

| Time/min | A% | B% |
| --- | --- | --- |
| 0 | 90 | 10 |
| 5 | 90 | 10 |
| 15 | 75 | 25 |
| 30 | 65 | 35 |
| 40 | 50 | 50 |
| 45 | 90 | 10 |

(4)

| Time/min | A% | B% |
| --- | --- | --- |
| 0 | 95 | 5 |
| 5 | 95 | 5 |
| 15 | 90 | 10 |
| 35 | 85 | 15 |
| 40 | 95 | 5 |

(5)

| Time/min | A% | B% |
| --- | --- | --- |
| 0 | 95 | 5 |
| 5 | 95 | 5 |
| 20 | 92 | 8 |
| 45 | 88 | 12 |
| 50 | 95 | 5 |

(6)

| Time/min | A% | B% |
| --- | --- | --- |
| 0 | 95 | 5 |
| 5 | 95 | 5 |
| 35 | 90 | 10 |
| 40 | 95 | 5 |

**II. Supplementary Figures**

**Figure S1 | Representative chromatograms of the screening of chiral RP columns.** The chromatography was conducted using different chiral RP columns with the elution procedures listed in Supplementary Methods. The flow rate of 0.5 ml/min was used to couple with UV detection at 343 nm at 25℃. A, Chiralpak AS-RH with supplementary methods (1); B, Chiralcel OX-RH with supplementary methods (1); C, Chiralpak AY-RH with supplementary methods (2); D, Chiralcel OJ-RH with supplementary methods (6); E, Chiralpak AZ-RH with supplementary methods (3); F, Chiralcel OZ-RH with supplementary methods (5); G, Chiralpak AD-RH with supplementary methods (4).

**Figure S2 |** **A)** **The effects of isopropanol proportion on the enantiomeric resolution of Rac-HCQ**. **B) The effects of isopropanol proportion on the retention time of HCQ enantiomers**. Rt1 is the retention time of R-HCQ; Rt2 is the retention time of S-enantiomer. Chromatographic conditions: n-hexane containing 0.5% DEA at a flow rate of 1.0 ml/min with UV detection at 343 nm at 25℃.

**Figure S3 |** **Effects of DEA concentration on the enantiomeric resolution of Rac-HCQ**. Chromatographic conditions: Chiralpak AD-H (4.6 mm×150 mm, 5 μm particle size); n-hexane-isopropanol (93:7, v/v) with various concentrations of DEA in hexane as mobile phase at a flow rate of 1.0 ml/min with UV detection at 343 nm at 25℃.

**Figure S4 |** **Chromatogram of the resolution of racemic HCQ based on reported method.** Chromatogram of the resolution of racemic HCQ based on reported method. Chromatographic conditions: Chiralpak AD-H (4.6 mm×150 mm, 5 μm particle size); n-hexane-isopropanol (92:8, v/v) containing 0.1% DEA as mobile phase at a flow rate of 1.0 ml/min.

**III. Supplementary Equations**

The linear regression curves between peak area and sample concentration were obtained by utilizing Rac-HCQ samples ranging from 1 to25 μg/mL. Three standard curves of Rac-, S- and R-HCQ were plotted respectively, and the linear equation and the square value of the correlation coefficient (R^2^) were obtained. The equation for Racemate was $y=21801x-1927.9$ (R^2^ = 0.9955); the equation for S-enantiomer was $y=13173x-4205$ (R^2^ = 0.9968); the equation for R-enantiomer was $y=13331x-3378$ (R^2^ = 0.9973).

**IV. Supplementary Results**

**Irreproducibility and poor resolution by reported method**

Previously, two studies have reported the separation of two enantiomers of HCQ with Chiralpak AD-RH column using n-hexane and isopropanol in the ratio of 92:8 (v/v) containing 0.1% diethylamine (DEA) as mobile phase. Firstly, Chiralpak AD-RH is a reversed phase column, which is not compatible to this mobile phase. Therefore, we recognized that the column used in the literatures could actually be Chiralpak AD-H. Next, when reported chiral HPLC conditions were used with Chiralpak AD-H column, the resolution of racemic HCQ was extremely poor as shown in Figure S4.

**Unsuitability of chiral RP-HPLC columns**

Typically, reversed phase chromatography exhibits several advantages, including better resolution, shaper peaks and aqueous based mobile phase. Thus, to develop a suitable HPLC method for baseline separation of the enantiomers of HCQ, different mobile phases and stationary phases for reversed phase HPLC (RP-HPLC) were employed and compared. Various reversed phase chiral columns, such as Chiralcel OJ-RH, Chiralcel OX-RH, Chiralcel OZ-RH, Chiralpak AD-RH, Chiralpak AS-RH, Chiralpak AY-RH, Chiralpak AZ-RH, were screened for possible resolution of HCQ enantiomers. Unfortunately, none of the columns at the experimental conditions gave meaningful resolution (Fig. S1). As a result, reversed phase chiral HPLC was unlikely useful for the separation and quantification of HCQ enantiomers, which might be due to the fact that the chiral center of HCQ is located in the middle of the molecule with a large steric hindrance to prevent distinct recognition and binding between different enantiomers and the stationary phase.
